# A Novel Theoretical Expression for the Impedance of a Ferrite-Loaded CW Illuminator

**DOI:** 10.3390/s25175285

**Published:** 2025-08-25

**Authors:** Peng Chen, Yangzhen Qin, Fulin Wu, Guangshuo Zhang, Qi Xu, Tianao Li, Hongmin Lu

**Affiliations:** School of Electronic Engineering, Xidian University, Xi’an 710071, China; 1702310030@stu.xidian.edu.cn (P.C.); 22021110257@stu.xidian.edu.cn (Y.Q.); fulinwu@stu.xidian.edu.cn (F.W.); zhangguangshuoemc@stu.xidian.edu.cn (G.Z.); 23021211636@stu.xidian.edu.cn (Q.X.); 23021211957@stu.xidian.edu.cn (T.L.)

**Keywords:** CW illuminator loop antenna, ferrite-loaded, antenna theory, input impedance

## Abstract

The continuous-wave (CW) illuminator, whose fundamentals are related to the theoretical understanding of loop antennas loaded with ferrite materials, is a device which plays an important role in electromagnetic pulse (EMP) susceptibility assessment. However, existing theoretical formulas do not consider cases where ferrite materials are loaded into the loop antenna. This paper provides a new explicit theoretical formula for the impedance of a circular loop antenna loaded with ferrite materials for CW illuminator design, and explores the variation regularity of its input impedance. Loading ferrite materials affects the internal impedance of the loop antenna and forces some modifications to the classical calculation procedure, resulting in an asymptotic numerical calculation method and a closed-form solution. The full-wave simulation results from CST Studio Suite show a maximum error of less than 0.99%, compared to the classical theory. With ferrite material loaded, the input impedance of the loop antenna is significantly reduced and smoothed in a wide range of normalized radii. For a loop antenna with a fixed circumference, the input impedance indicates that the Q-factor decreases as the thickness of the ferrite material increases. Conversely, for a ferrite-loaded loop antenna with a constant material thickness, a larger loop circumference results in a higher Q-factor. In summary, this study provides a fast and accurate computational method for the input impedance design of CW illuminators, while also offering an effective tool for further research on the performance of ferrite-loaded loop antennas.

## 1. Introduction

Loop antennas and their various modifications are widely used in applications such as UHF RFID [1,2,3], brain lobe imaging/millimeter-wave communication [4,5], wireless power transfer [6,7], and remote sensing/geophysical exploration [8,9,10]. In the 1950s and the 1960s, the fundamental characteristics of loop antennas were established, respectively, by Storer [11] and Wu [12], who derived analytical expressions for the impedance and current distribution of unloaded thin-wire loops. After that, K.Izuka studies the input impedance of a circular loop loaded with arbitrary number of lumped impedances spaced at equal intervals [13], however the loop wire is assumed as a perfect conductor and the surface impedance is ignored. For the next few decades, minimal attention has been paid to loops working at radio and microwave frequency band, compared to other types of antennas, until the appearance of metamaterial [14]. In order to study properties of antennas res-onant at high frequencies, a large collection of literatures has focused on the subject of extending antenna theory to infrared and optical regions [15,16,17].

Building upon Storer and Wu’s framework, McKinley [18] introduced a modified formulation that incorporated the surface impedance of metallic wires, enabling accurate impedance characterization even at optical frequencies. Subsequently, the same approach was applied to extend Izuka’s theory to infrared/optical regimes for loops with periodic lumped loads [19]. However, the low-frequency characteristics of the loaded loop antenna have not received sufficient attention. Recently, a specialized loop antenna, referred to as the CW illuminator, has been revisited, and a fast numerical simulation method has been proposed for calculating its field distribution [20,21]. However, this work does not account for the theoretical model and the closed-form solution of the impedance.

This paper presents a novel analytical model for characterizing the input impedance of a ferrite-loaded circular loop antenna. A closed-form solution for the input impedance is derived and subsequently validated through full-wave electromagnetic simulations using three-dimensional field analysis software. The proposed model enables systematic investigation of impedance variation patterns under different loading conditions. Furthermore, this work provides an efficient and accurate design methodology for optimizing impedance characteristics in continuous-wave (CW) radiator applications.

## 2. Review of the Classical Theory of a Circular Loop

### 2.1. The Origin of the Problem

**Figure 1:**
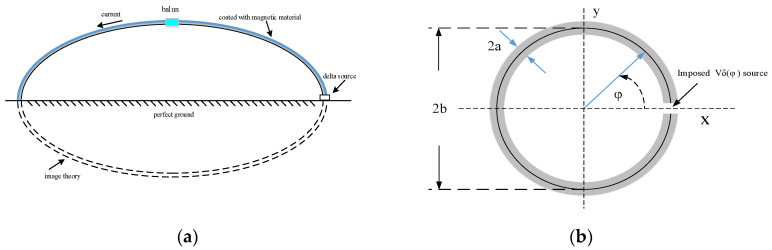
Geometries of (**a**) a CW illuminator, considering image theory; and (**b**) a classical loop.

Figure 1a illustrates the complete equivalent geometry of a continuous-wave (CW) illuminator, which consists of a semi-elliptical structure with a delta-function source placed on the ground plane and coated with a ferrite material. In [22], the magnetic medium is initially modeled as a continuous ferrite tube enclosing the wire which is subsequently simplified to discrete ferrite beads loaded along the conductor [23]. In this work, the structure is modeled by assuming that the elliptical surface is uniformly coated with a magnetic film. From a theoretical perspective, on the basis of image theory, the circular loop shown in Figure 1b provides a reasonable approximation of the CW illuminator configuration shown in Figure 1a, provided that both loops enclose the same area.

### 2.2. The Impedance of a Circular Loop Without Loads

The theoretical analysis begins with a thin-wire circular loop, as previously studied by Storer [11] and Wu [12]. Figure 1b illustrates the geometry and coordinate system of a metallic loop with an infinitesimal gap. The loop has a radius b and a wire radius a, and it is excited by a delta-function voltage source applied across the gap. When the condition a << b is satisfied, it is reasonable to assume that the surface current flows only in the azimuthal direction along the loop.

The well-known Pocklington integral equation establishes the relationship between the current distribution and the applied voltage source on an unloaded loop. It is expressed as follows [11]:

(1) $$V\delta \left(\varphi \right)=\frac{j\zeta_{0}}{4\pi }\int_{-\pi }^{\pi }K\left(\varphi -\varphi^{\prime }\right)I\left(\varphi^{\prime }\right)d\varphi^{\prime }$$

where $I\left(\varphi^{\prime }\right)$ is the total current at φ on the loop; V is the voltage at φ=0; $\delta \left(\varphi \right)$ is the Dirac delta function; $\zeta_{0}=\sqrt{\frac{\mu_{0}}{\varepsilon_{0}}}=120\pi$Ω is the wave impedance in free space; and $K\left(\varphi -\varphi^{\prime }\right)$ is the kernel of Equation (1), which is given by

(2) $$K\left(\varphi -\varphi^{\prime }\right)=\left[kb\cos\left(\varphi -\varphi^{\prime }\right)+\frac{1}{kb}\frac{\partial^{2}}{\partial \varphi^{2}}\right]\cdot \frac{\exp\left(-jkbR\left(\varphi -\varphi^{\prime }\right)\right)}{R\left(\varphi -\varphi^{\prime }\right)}$$

(3) $$R\left(\varphi -\varphi^{\prime }\right)={\left[4\sin^{2}\left(\frac{\varphi -\varphi^{\prime }}{2}\right)+a^{2}/b^{2}\right]}^{1/2}$$

where a is the radius of the wire; b is the radius of the loop; and k=2π/λ;

The infinite Fourier series is employed to describe the current distribution. This can be written as

(4) $$I\left(\varphi \right)=\sum_{n=-\infty }^{\infty }I_{n}\exp(jn\varphi )=\frac{V\delta \left(\varphi \right)}{j\pi \zeta_{0}}\left[\frac{1}{a_{0}}+2\sum_{n=1}^{\infty }\frac{\cos(n\varphi )}{a_{n}}\right]$$

where $\zeta_{0}$, $\delta \left(\varphi \right)$ is as defined above; and $a_{0}\cdot \cdot \cdot a_{n}$ are the coefficients related to the integrand in [11]. The loop input impedance at φ=0 is defined as

(5) $$Z=\frac{V\delta \left(\varphi \right)}{I\left(\varphi \right)}=\frac{j\pi \zeta_{0}}{\left[\frac{1}{a_{0}}+2\sum_{n=1}^{\infty }\frac{\cos(n\varphi )}{a_{n}}\right]}$$

### 2.3. The Impedance of a Circular Loop Loaded with Ferrite Material

The cylindrical antenna with spatially varying internal impedance was analyzed by Wu [24]. The antenna is composed of a resistive material whose conductivity varies along its length. As current flows along the structure, the resistive material imposes a distributed opposition, effectively forming an internal impedance within the antenna.

In the present study, a circular loop antenna coated with ferrite material is considered. Based on the formulations developed by Wu and Storer [11,24,25], the current distribution on the ferrite-loaded circular loop can be expressed as follows:

(6) $$Z_{f}I\left(\varphi \right)=V\delta \left(\varphi \right)-\frac{j\zeta_{0}}{2}\sum_{n=-\infty }^{\infty }a_{n}I_{n}\exp(jn\varphi )$$

where $Z_{f}$ is the internal impedance caused by the loaded ferrite material in low frequency. Inserting (4) into (6) leads to

(7) $$V\delta \left(\varphi \right)=\sum_{n=-\infty }^{\infty }\left(\frac{j\zeta_{0}}{2}a_{n}+Z_{f}\right)I_{n}\exp(jn\varphi )$$

Integration on both sides of Equation (7) reduces it to

(8) $$\begin{array}{l} \frac{1}{2\pi }\int_{-\pi }^{\pi }V\delta \left(\varphi \right)\exp(-jn\varphi )d\varphi \\ =\frac{1}{2\pi }\int_{-\pi }^{\pi }\sum_{m=-\infty }^{\infty }\left(\frac{j\zeta_{0}}{2}a_{m}+Z_{f}\right)I_{m}\exp(jm\varphi )\exp(-jn\varphi )d\varphi \\ I_{n}=\frac{V}{2\pi \left(\frac{j\zeta_{0}}{2}a_{n}+Z_{f}\right)} \end{array}$$

Inserting (8) into (4) reduces it to

(9) $$\begin{array}{l} I\left(\varphi \right)=\sum_{n=-\infty }^{\infty }I_{n}\exp(jn\varphi )\\ =\sum_{n=-\infty }^{\infty }\frac{V}{2\pi \left(\frac{j\zeta_{0}}{2}a_{n}+Z_{f}\right)}\exp(jn\varphi )\\ =V\left[\frac{1}{j\pi \zeta_{0}a_{0}+2\pi Z_{f}}+\sum_{n=1}^{\infty }\frac{\cos(jn\varphi )}{\frac{j\pi \zeta_{0}}{2}a_{n}+\pi Z_{f}}\right] \end{array}$$

The impedance at the gap then becomes

(10) $$\begin{array}{l} Z=\frac{V}{I\left(\varphi =0\right)}=1/\left[\frac{1}{j\pi \zeta_{0}a_{0}+2\pi Z_{f}}+\sum_{n=1}^{\infty }\frac{1}{\frac{j\pi \zeta_{0}}{2}a_{n}+\pi Z_{f}}\right]\\ =1/\left[\frac{1}{Z_{0}^{\prime }}+\sum_{n=1}^{\infty }\frac{1}{Z_{n}^{\prime }}\right] \end{array}$$

where

(11) $$Z_{0}^{\prime }=j\pi \zeta_{0}a_{0}+2\pi Z_{f}$$

(12) $$Z_{n}^{\prime }=j\pi \zeta_{0}\left(a_{n}/2\right)+\pi Z_{f}$$

## 3. Analysis of the Internal Impedance

### 3.1. The Dispersion Characteristic of Ferrites

**Figure 2:**
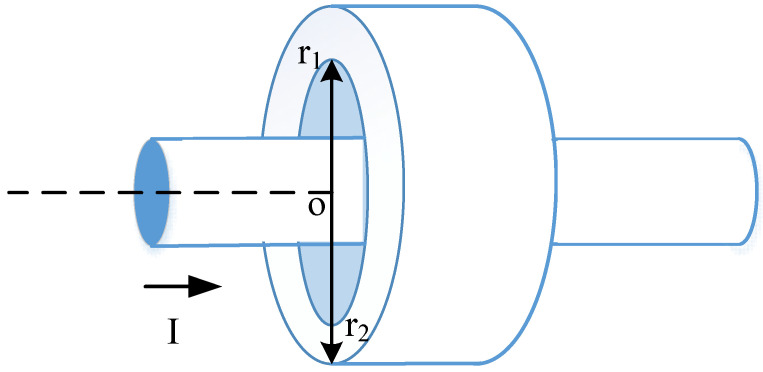
The interaction between ferrite and the loop wire.

The impedance of the ferrite-loaded loop is expressed in (10), where the internal impedance term *Z*_f_ requires further derivation. The interaction between ferrite and the loop wire at low frequencies—where ferrite materials exhibit significant magnetic activity—may be illustrated as in Figure 2. In this frequency range, the current flowing through the loop is reduced due to two primary mechanisms: energy is consumed in establishing the magnetic flux within the ferrite material, and additional energy is dissipated as losses within the ferrite itself.

The impedance of ferrite beads presented to a loop is expressed as in [26], as follows:

(13) $$Z_{f}=j\omega \left[\frac{\mu_{0}}{2\pi }\ln\left(\frac{r_{2}}{r_{1}}\right)\right]\mu_{r}\cdot 2\pi b$$

where *r*_1_ and *r*_2_ are the inner radius and outer radius, respectively, of a ferrite bead loading on a loop, and *μ*_r_ is the relative permeability.

Because the impedance of a ferrite bead is directly influenced by its magnetic permeability characteristics, it is essential to investigate the underlying magnetization mechanisms and the frequency-dependent dispersion behavior of permeability. Extensive research has been conducted on magnetic materials, particularly Ni-Zn and Mn-Zn ferrites [27,28].

As reported by Takanori in [27], two distinct types of magnetic resonance appear in the permeability spectra of Ni-Zn and Mn-Zn ferrites. These resonances are attributed to domain-wall motion and spin rotation, respectively. By superimposing the contributions from both mechanisms, the complex relative permeability of these ferrites can be modeled as follows:

(14) $$\mu_{r}=1+\chi_{d}+\chi_{s}=1+\frac{\omega_{d}^{2}\chi_{d0}}{\omega_{d}^{2}+\omega +i\omega \beta }+\frac{\left(\omega_{s}+i\omega \alpha \right)\omega_{s}\chi_{s0}}{{\left(\omega_{s}+i\omega \alpha \right)}^{2}-\omega^{2}}$$

The parameters are as follows:

- $\chi_{d}$ domain-wall motion magnetic susceptibility.
- $\chi_{s}$ spin motion magnetic susceptibility.
- $\chi_{d0}$ static magnetic susceptibility for domain-wall component.
- $\chi_{s0}$ static magnetic susceptibility for spin component.
- $\omega_{d}$ resonance frequency of domain-wall component.
- $\omega_{s}$ resonance frequency of spin component.

Inserting Equation (14) into (13) leads to

(15) $$Z_{f}=j\omega \left[\frac{\mu_{0}}{2\pi }\ln\left(\frac{r_{2}}{r_{1}}\right)\right]\left(1+\frac{\omega_{d}^{2}\chi_{d0}}{\omega_{d}^{2}+\omega +i\omega \beta }+\frac{\left(\omega_{s}+i\omega \alpha \right)\omega_{s}\chi_{s0}}{{\left(\omega_{s}+i\omega \alpha \right)}^{2}-\omega^{2}}\right)\cdot 2\pi b$$

The ferrite model parameters in [26] are illustrated in Table 1, and the Mn-Zn parameters are adopted for theory analysis.

### 3.2. Comparison of the Surface and Ferrite Impedance

The surface impedance of a cylindrical conductor becomes negligible at low frequencies, but it becomes significant in the optical frequency range. In contrast, the impedance contribution from ferrite materials is dominant at low frequencies and diminishes as the frequency increases. These distinct impedance behaviors are primarily governed by the permittivity of the conductor [18] and the permeability of the ferrite, respectively.

According to the literature [28,29], elastic oscillations and interband transitions of the electron gas in metals both contribute to frequency-dependent permittivity. These mechanisms are effectively captured by analytical models. Similarly, in ferrite materials, the motion of magnetic domains exhibits behavior analogous to that of electron gas in metals. Domain-wall motion corresponds to elastic oscillation, while spin dynamics and resonance bandwidth transitions share a similar mathematical representation [27].

**Figure 3:**
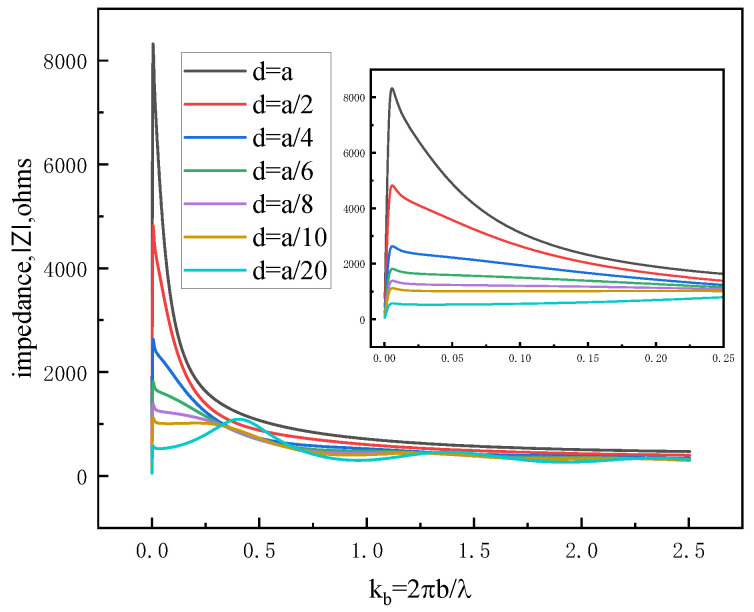
Magnitude of impedance as a function of normalized radius k_b_ for various ferrite thicknesses d.

As a result, the impedance characteristics of a ferrite-loaded circular loop become particularly prominent at low frequencies. The introduction of ferrite material leads to a smoother overall impedance profile and reduces the input impedance of the antenna, thereby facilitating improved impedance matching. Figure 3 shows the magnitude of ferrite-loaded loop impedance varying with the variable d, where d is the thickness of the loaded ferrite material. The results are also plotted against the independent variable $k_{b}=2\pi b/\lambda$, where λ is the exciting wavelength. An increase in $k_{b}$ can be viewed in two ways: (1) as an increase in the size of the loop circumference, given a constant frequency; or (2) as an increase in the excitation, given a constant circumference. The waveform details at low frequencies for $k_{b}\in \left(0,0.25\right)$ are shown in the subgraph of Figure 3. The results show that for a ferrite-loaded loop antenna with a fixed thickness ratio of *b*/*a* = 64, the magnitude of the input impedance gradually decreases and becomes smoother as *d* is reduced.

**Figure 4:**
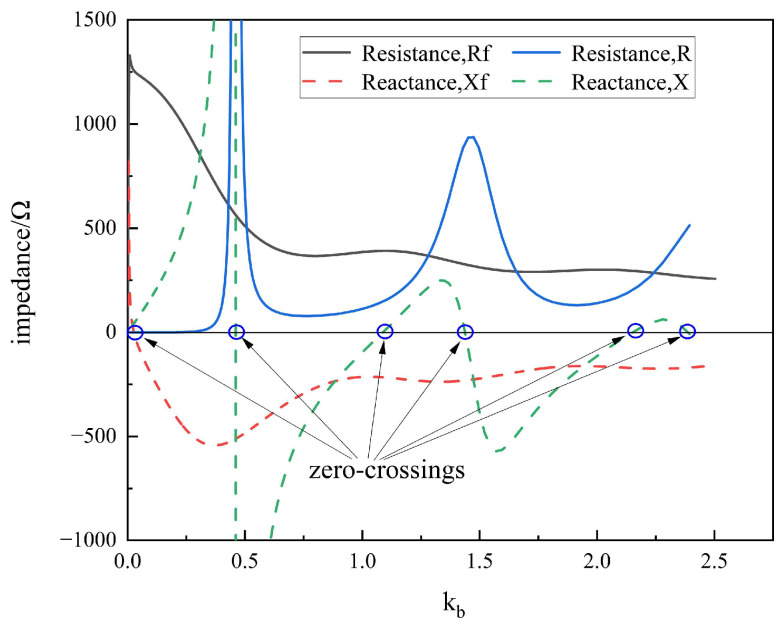
Comparison of Q factor between loops with and without ferrite loads.

Ferrite materials can also impact the Q factor of the loop. Figure 4 illustrates a comparison of two impedance characteristics, where Rf and Xf are the resistance and reactance of the impedance of the loaded loop, while R and X are the unloaded ones. The zero-crossings occur when the loop resonates. When the loop is not loaded, every mode resonates in a narrow band. This means the modes do not interface each other; that is, the Q factors of these modes, as measured by the mode central resonances divided by the mode bandwidth, are quite high. On the other hand, as shown in Figure 4, the bandwidth of every mode in a ferrite-loaded loop is so broadened that the resonance curves overlap each other, and the remaining resonances (except the first) disappear entirely. This phenomenon indicates that the Q factor of the ferrite-loaded loop is very low.

## 4. Validations of the Proposed Impedance Formulation

### 4.1. Evaluation of the Loop Impedance with Ferrite Loads

For loops without ferrite loading, the impedance is given by Equation (4) above. Storer [11] provided an approximate expression for the parameter $a_{n}$ when the mode number *n* > 5, as follows:

(16) $$a_{n}\approx \frac{1}{\pi }\left(k_{b}-\frac{n^{2}}{k_{b}}\right)\left(\ln\left(\frac{2b}{a}e^{-0.5772}\right)-\ln\left(n\right)-j\times \frac{{\left(k_{b}\right)}^{2n+1}}{\Gamma \left(2n+2\right)}\right)n>k_{b},n\gg 1$$

He subsequently replaced the summation over terms for *n* > 5 with an integral representation, and introduced a parameter $\Omega_{T}=2\ln(2\pi b/a)$ to define the thickness of loops. This parameter is related to the loop structure a, b and can be used to distinguish different size of loops. Following a series of analytical derivations, the integral for loops with different thickness was evaluated as follows:

(17) $$\Psi =2\sum_{n=5}^{\infty }\frac{\cos(n\varphi )}{a_{n}}=\frac{2\pi }{\ln\left(\frac{n_{0}}{4.5}\right)}\frac{k_{b}}{4.5}\left[J+\frac{1}{3}{\left(\frac{k_{b}}{4.5}\right)}^{2}\right]$$

where $J=\left(0.47,0.9,1.25,1.4,1.4\right)$ for $\Omega_{T}=\left(8,9,10,11,12\right)$; and $n_{0}=2b/a\exp(-0.5772)$.

The impedance expression in (10) is analytically complex and not easily evaluated through closed-form derivation. To approximate the impedance, a numerical computation approach is employed. Given that the current on the loop is continuous and bounded, the resulting series is convergent. Furthermore, as n increases, the summation over terms with *n* > 5 approaches a constant function that depends solely on the product k_b_. Accordingly, the impedance can be reformulated as

(18) $$Z=\frac{V}{I\left(\varphi =0\right)}=1/\left[\frac{1}{Z_{0}^{\prime }}+\sum_{n=1}^{4}\frac{1}{Z_{n}^{\prime }}+\psi \left(k_{b}\right)\right]$$

Given that the first five terms in (17) are explicitly known, the remaining task is to estimate the residual sum. According to the Euler–Maclaurin formula, the summation can be approximated by an integral, which yields the following expression:

(19) $$\psi \left(k_{b}\right)=\sum_{n=5}^{N}f\left(n\right)=\sum_{n=5}^{N}\frac{1}{Z_{n}^{\prime }}\approx \int_{5}^{N}f\left(x\right)dx+\frac{\left(f\left(5\right)+f\left(N\right)\right)}{2},N\to \infty$$

Substituting (12) and (15) into (18), and reducing, we obtain the following:

(20) $$\begin{array}{l} \psi \left(k_{b}\right)\approx \int_{5}^{N}\frac{1}{j\pi \zeta_{0}\left(\frac{1}{\pi }\left(k_{b}-\frac{n^{2}}{k_{b}}\right)\left(\ln\left(\frac{2b}{an}e^{-0.5772}\right)-j\times \frac{{\left(k_{b}\right)}^{2n+1}}{\Gamma \left(2n+2\right)}\right)/2\right)+\pi Z_{f}}dx\\ +\frac{f\left(N\right)+f\left(5\right)}{2}\\ \approx \psi_{0}+\frac{f\left(5\right)}{2} \end{array}$$

where a and b are the wire radius and loop radius, respectively; and $k_{b}=2\pi b/\lambda$.

**Figure 5:**
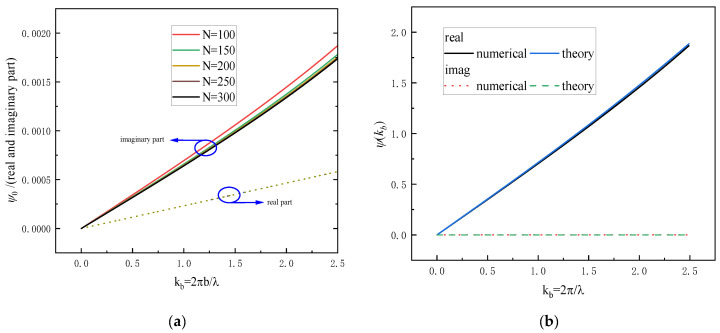
Numerical calculations of $\psi_{0}$ for (**a**) a loop loaded with ferrite; and (**b**) a loop without loads.

A numerical program was implemented to compute the first term in (20), $\psi_{0}$, for *N* = 100, 150, 200, 250 and 300. Figure 5a illustrates the asymptotic behavior of the real and imaginary components of $\psi_{0}$ as *N* increases. A comparison between the numerical results and the analytical expression given in (16) is presented in Figure 5b, where the relative error is observed to be less than 0.99%.

It can be seen from Figure 5a that both the real and imaginary components of $\psi_{0}$ exhibit approximately linear behavior. The imaginary component intersects the points (0, 0) and (2.5, 1.7 × 10^3^, while the real component passes through (0, 0) and (2.5, 5.8 × 10^4^). Based on these observations, the approximate linear expression for $\psi_{0}$ can be derived as follows:

(21) $$\psi \left(k_{b}\right)\approx \frac{\left(0.58+j1.7\right)}{2.5}\times 10^{-3}k_{b}+\frac{\left(f\left(\infty \right)+f\left(5\right)\right)}{2}$$

### 4.2. Comparison Between Theoretical and Simulated Impedance of the Ferrite-Loaded Loop

**Figure 6:**
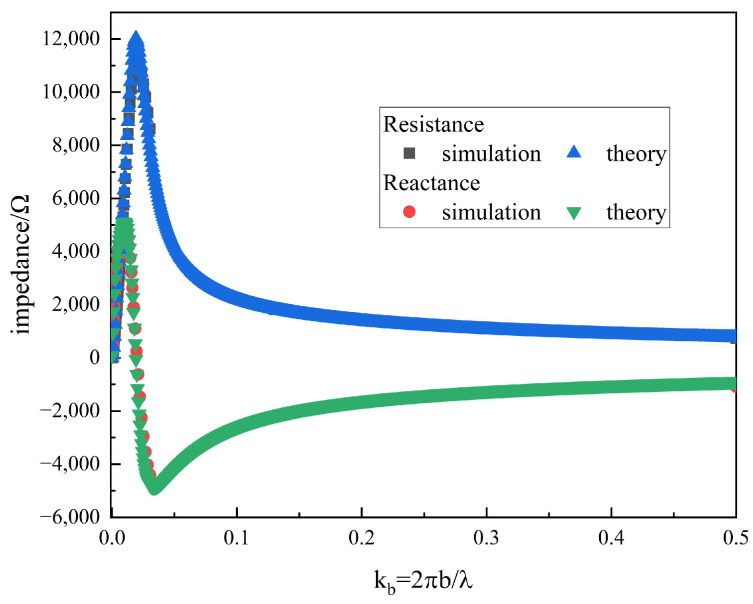
Comparison of results from simulation and theory, with $\Omega_{T}=2\ln(\frac{2\pi b}{a})=12$.

To validate the closed-form impedance expressions proposed in this study, numerical results were compared with full-wave simulations performed using CST Microwave Studio. The input impedance of the ferrite-loaded loop antenna obtained from the analytical solution was compared with the results from CST simulation, as illustrated in Figure 6, where excellent agreement between the two can be observed. The loop antenna has a radius of 304.8 mm and a wire diameter of 4.8 mm, while the ferrite material exhibits a thickness of 0.625 mm. Due to the limited accuracy of the time-domain solver in CST for low-frequency problems, the frequency-domain solver was employed during the validation process.

The parameters of Mn-Zn Ferrite given in Table 1 were adopted to carry out the simulation. The simulation was performed using CST 2023 on a HP Z480 workstation. First, the complex permeability of the Mn-Zn ferrite material in the frequency range of 10 kHz to 150 MHz was calculated using Equation (12), with a frequency step of 10 kHz. Next, the permeability material data were configured in CST. The computed discrete frequency-permeability data were imported via the path “Material properties->Dispersion->Magnetic Dispersion->User->Dispersion List->Load File,” allowing CST to perform fitting and interpolation of the ferrite material’s permeability based on the imported data. Finally, a discrete port was employed in CST to excite the loop antenna model. Upon completion of the simulation, the input impedance of the loop antenna loaded with the ferrite material was calculated using the post-processing function in CST.

## 5. Conclusions

The characteristics of an unloaded thin-wire loop antenna can be accurately described using classical theory. However, for specific applications, it becomes necessary to load the antenna with ferrite material and derive a theoretical expression for its input impedance. In this work, a new impedance expression is proposed by introducing an additional term to the unloaded case. Comparisons with CST simulation results are conducted to validate the theoretical model. Overall, the proposed expression shows good agreement with simulation in the range 0 < *k_b_* < 2.5. The results presented in this paper can be utilized for rapid computation of the input impedance of ferrite-loaded loop antennas and for the design of impedance-matching networks.

The ferrite-loading influences the antenna impedance by affecting the current distribution. The underlying physical mechanisms include spin rotation and magnetic domain-wall motion, which contribute to the dispersive behavior of the ferrite material. The permeability data used in the CST simulations is based on a model proposed in [26].

## Figures and Tables

**Table 1:** Ferrite model parameters.

Ferrite Material	Density (g/cc)	Domain-Wall Component			Spin Component		
		$\chi_{d0}$	$\omega_{d}$ (MHz)	β	$\chi_{s0}$	$\omega_{s}$ (MHz)	α
Mn-Zn Ferrite	4.90	3282	2.5	9.3 × 10^6^	1438	6.3	1.28
Ni-Zn Ferrite	5.20	485	2.8	3.5 × 10^6^	1130	1100	161

## Data Availability

The datasets generated and analyzed during the current study are not publicly available but are available from the corresponding author on reasonable request.
